# Construction and Periplasmic Expression of the Anti-EGFRvIII ScFv Antibody Gene in *Escherichia coli*

**DOI:** 10.3797/scipharm.ISP.2015.06

**Published:** 2016-02-14

**Authors:** Kartika Sari Dewi, Debbie Sofie Retnoningrum, Catur Riani, Asrul Muhamad Fuad

**Affiliations:** 1 Research Center for Biotechnology, Indonesian Institute of Sciences (LIPI), Jalan Raya Bogor Km. 46, 16911, Cibinong, Bogor, Indonesia; 2 School of Pharmacy, Bandung Institute of Technology, Jalan Ganesha 10, 40132, Bandung, Indonesia

**Keywords:** scFv, EGFRvIII, Periplasmic expression, PelB, *E. coli*

## Abstract

In the previous study, we constructed an expression vector carrying the anti-EGFRvIII scFv antibody gene with V_H_-linker-V_L_ orientation. The proteins were successfully produced in the periplasmic space of *Escherichia coli*. In this study, we substituted the inserted DNA with V_L_-linker-V_H_ orientation of the anti-EGFRvIII scFv gene and analyzed its expression in *E. coli*. The DNA fragment was amplified from its cloning vector (pTz-rscFv), subsequently cloned into a previous expression vector containing the pelB signal sequence and his-tag, and then transformed into *E. coli* TOP10. The recombinant plasmids were characterized by restriction, PCR, and DNA sequencing analyses. The new anti-EGFRvIII scFv antibody proteins have been successfully expressed in the periplasmic compartment of *E. coli* Nico21(DE3) using 0.1 mM final concentration of IPTG induction. Total proteins, soluble periplasmic and cytoplasmic proteins, solubilized inclusion bodies, and extracellular proteins were analyzed by SDS-PAGE and Western Blot analyses. The results showed that soluble scFv proteins were found in all fractions except from the cytoplasmic space.

## Introduction

Antibodies have crystallizable fragment (Fc) domain which binds to Fc receptors and recruits cytotoxic effector molecules and, by interacting with the neonatal Fc receptor, provides long serum half-lives [1, 2]. However, in therapeutic application, there are situations in which the Fc-mediated effects are not required or even undesirable. Thus, with the latest technology, a wide variety of genetically engineered antibodies have been produced, including single-chain Fv (scFv) antibodies in which the genes of variable heavy (V_H_) and variable light (V_L_) domain joined together with short peptide linker or disulfide bond [3].

In comparison to the parental antibodies, scFv antibodies have several advantages in clinical practice including better tumour penetration, more rapid blood clearance and lower retention times in non-target tissue, which may be beneficial in radiotherapy and diagnostic applications. Also, the lack of Fc region in scFv antibodies leading to low immunogenicity, making them better therapeutic agents for many applications [4, 5]. One of therapeutic application of scFv is as a targeting moiety in targeted cancer therapy. In such application, scFv must have a highly specific target which is not found in normal cells.

Epidermal growth factor receptor variant III (EGFRvIII) is a mutant variant of EGFR that commonly overexpressed in human malignant cells. EGFRvIII has in frame deletion of exons 2–7 that encodes extracellular domain, resulting in the formation of new Glycine residue. EGFRvIII is commonly found in glioblastoma multiforme and also been reported in breast, ovarian, prostate, lung, head and neck carcinomas, but is not found in normal cells [6–8]. Lacks of 267 amino acids from extracellular domain of EGFRvIII has resulted in the formation of a new immunogenic epitope near the amino terminus. Therefore, this receptor might be used as an ideal molecular target in immunology-based cancer therapy [9].

Based on its molecular weight, scFv antibody can be easily expressed in *Escherichia coli* and allowing protein engineering to improve its properties, such as increase of affinity and alteration of specificity [10]. However, scFv molecule has at least two disulfide bonds in its structure which are required to be correctly formed in order to preserve its antigen-binding affinity. In *E. coli* expression system, periplasmic compartment is preferably used for recombinant protein production that needs a correct protein folding and disulfide bond formation [11].

Good expression levels of scFv antibodies in *E. coli* can be achieved via soluble production, secreting scFv into the oxidizing environment of the bacterial periplasm where assembly and formation of disulfide bond can occur. Periplasmic secretion is achieved by genetically fusing the signal sequence onto the N-terminus of the scFv sequence. In this study, we used pelB signal sequence which is derived from *Erwinia carotovora* [12]. This signal sequence consists of 22 amino acids residues, which leads the scFv expression product into the periplasmic compartment in *E. coli* [11, 13].

Kim et al. have shown that the expression of scFv antibody against c-Met in *E. coli* expression system is significantly influenced by the orientation of its variable domains. However, the purified scFv antibodies of the two different formats exhibited almost the same antigen-binding activities [14]. In contrast, Desplancq et al. [15] reported that scFvs of the tumour-binding *antibody B72.3* with the orientation V_L_-linker-V_H_ showed greater binding activity than those with the orientation V_H_-linker-V_L_.

Our previous study has successfully constructed and expressed an anti-EGFRvIII scFv antibody gene with V_H_-linker-V_L_ orientation in *E. coli* periplasm [16]. Until now, no report has been found concerning expression of an anti-EGFRvIII scFv with reversed orientation in *E. coli*. In order to determine the periplasmic expression of an anti-EGFRvIII scFv with reversed orientation, we changed the orientation of previous scFv gene from V_H_-linker-V_L_ into V_L_-linker-V_H_. And then, we substituted the insert DNA from previous expression vector with V_L_-linker-V_H_ orientation of scFv (rscFv) gene and expressed it in *E. coli* Nico21(DE3).

## Results and Discussion

**Fig. 1:**
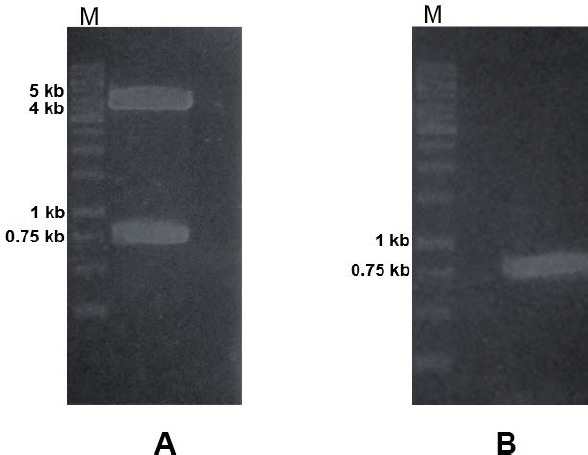
Confirmation of plasmids using restriction analysis. Previous expression plasmid (pJ414-scFv) (A) and amplified rscFv gene (B) were digested with *Nco*I and *Cla*I restriction enzymes. The digested plasmid was analyzed using agarose gel electrophoresis.

**Fig. 2:**
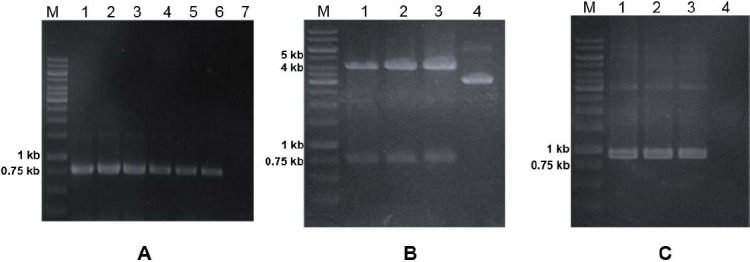
(A) Colony PCR analysis of transformants *E. coli* TOP10 using rscFv-F and rscFv-R primers. Lane 1-5, amplification fragment from colony number 1-5 (respectively); lane 6, positive control; lane 7, negative control. (B) Restriction analysis of pJ414-rscFv using *Nco*I and *Cla*I enzymes. Lane 1-3, pJ414-rscFv from colony number 1-3 (respectively); lane 4, uncut pJ414-rscFv. (C) PCR analysis of pJ414-rscFv using T7 promotor and rscFv-R primers. Lane 1-3, amplification fragment from colony 1-3 (respectively); lane 4, negative control.

Plasmid construction was performed to obtain the recombinant plasmid carrying rscFv gene. The rscFv gene was first amplified using pTz-rscFv as template and the result showed 0.75 kb of PCR product (Figure not shown). The expression vector was prepared by digestion of pJ414-scFv using *Nco*I and *Cla*I restriction enzymes (Fig. 1A). The rscFv gene was also digested using the same enzymes (Fig. 1B). The rscFv gene was inserted between pelB signal sequence and his-tag sequence. Growing transformants were characterized by colony PCR using rscFv-F and rscFv-R specific primers. Agarose gel electrophoresis showed a band with size of 0.75 kb (Fig. 2A).

To confirm correct recombinant plasmids, a number of 3 positive colonies were picked up and the plasmids were characterized by restriction, PCR and DNA sequencing analyses. The recombinant plasmid obtained was named pJ414-rscFv. Restriction analysis was carried out to determine the actual size of expression vector and insert DNA. Fig. 2B showed restriction of recombinant plasmids using *Nco*I and *Cla*I enzymes, resulting DNA bands with size of 0.75 kb and 4 kb which corresponded to the theoretical size of insert DNA and expression vector, respectively.

The PCR analysis was performed using T7-promoter and rscFv-R primers. These primers were used to determine the full-length construct of the gene within the pJ414-rscFv plasmid. Thus, the PCR product would consist of T7 promoter, pelB leader sequence, and rscFv gene. Fig. 2C showed a DNA band of approximately 0.9 kb, which corresponds to the theoretical size of desirable fragment. Based on DNA sequencing analysis (data not shown), there was no mutation in DNA encoding anti-EGFRvIII single-chain Fv antibody gene. After all of analyses conducted, it could be concluded that the recombinant plasmid was successfully constructed.

**Fig. 3:**
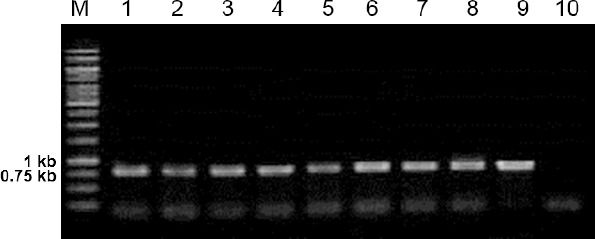
Colony PCR analysis of transformants *E. coli* Nico21(DE3) using rscFv-F and rscFv-R primers. Lane 1-8, amplification fragment from colony number 1-8 (respectively); lane 9, positive control; lane 10, negative control.

**Fig. 4:**
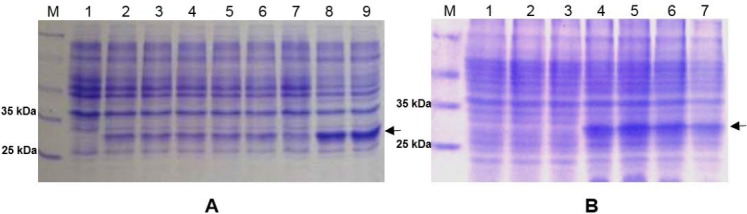
SDS-PAGE analysis of (A) Total proteins of *E. coli* Nico21(DE3) carrying pJ414-rscFv. Lane 1, total proteins from untransformed *E. coli* Nico21(DE3); lane 2–9, total proteins from colony number 1–8 (respectively), induced with 1 mM IPTG. (B) Total proteins of *E. coli* Nico21(DE3) induced with various IPTG concentration. Lane 1–2, untransformed *E. coli* Nico21(DE3) induced with 0 and 0.5 mM IPTG (respectively); lane 3–7, Transformant *E. coli* Nico21(DE3) from colony number 8 induced with 0, 0.1, 0.25, 0.5, and 1 mM IPTG (respectively). The rscFv band is marked with an arrow.

Confirmed pJ414-rscFv was transformed into *E. coli* Nico21(DE3) and characterized by colony PCR using rscFv specific primers (Fig. 3). Screening is important because not all colonies that arise following transformation may contain a plasmid with the desired DNA insert [17]. Agarose gel electrophoresis showed that all selected colonies gave positive results which can bee seen as a band with size of 0.75 kb (Fig. 3). In order to examine the expression of rscFv proteins, a single colony from every positive clone of *E. coli* Nico21(DE3) carrying pJ414-rscFv was cultured and induced with 1 mM IPTG. Characterization of total proteins using SDS-PAGE showed that good expression level obtained from colony number 7 and 8 (Fig. 4A).

The analysis was continued to determine the optimal concentration of IPTG as inducer. Fig. 4B lanes 3-7 showed total protein bands of induced *E. coli* culture using various IPTG concentrations. The result showed that there was no significant difference in the expression level of rscFv gene in the range of 0.1–1 mM of IPTG induction. Based on this result, low concentration of IPTG was used for rscFv expression. The rscFv band is deduced to have approximate size of 29.81 kDa as it is shown on the SDS-PAGE analysis (Fig. 4A and 4B). The band size corresponds to the theoretical size of rscFv proteins.

*E. coli* is currently the host of choice for producing antibody fragment. The oxidizing environment of periplasmic space, rich in proteins which are important for folding and catalyzing disulfide bond formation (PDI, DsbA and DsbC) or chaperones such as SKp [18, 19], is most suitable for proper folding of scFv fragment. Besides, extraction of periplasmic-expressed proteins can easily be performed by a simple osmotic shock procedure [20].

In order to reach the periplasm, the rscFv proteins are equipped with an N-terminal signal sequence that guides them to the Sec-translocon, which is a protein-conducting channel in cytoplasmic membrane [21]. In this study, pelB signal sequences was used to guide rscFv proteins into periplasmic space via post-translational SecB-targeting pathway. SecB, a cytosolic chaperone in *E. coli*, facilitates the export of pre-mature proteins by maintaining them in non-native conformation and passing them to SecA. SecA then acts in concert with SecYEG to translocate the proteins through the membrane to the periplasm [22].

**Fig. 5:**
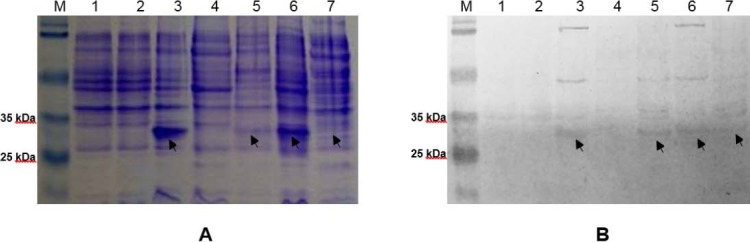
(A) SDS-PAGE and (B) Western blot analyses of rscFv gene expression in transformant *E. coli* Nico21(DE3) from colony number 8, induced with 0.1 mM IPTG. Lane 1, total proteins of untransformed *E. coli* Nico21(DE3). Lane 2, total proteins of uninduced transformant *E. coli* Nico21(DE3). Lane 3, total proteins of induced transformant *E. coli* Nico21(DE3). Lane 4, soluble cytoplasmic proteins fraction. Lane 5, soluble periplasmic proteins fraction. Lane 6, solubilized inclusion bodies. Lane 7, extracellular proteins fraction. The rscFv band is marked with an arrow.

A comparative analysis of total proteins, soluble cytoplasmic and periplasmic proteins, solubilized inclusion bodies, and extracellular proteins was carried out to find out the rscFv expression profile. Fig. 5A showed that rscFv protein bands were found in all fractions except from soluble cytoplasmic proteins fraction. A band of rscFv proteins from soluble periplasmic fraction was very thin. There was, however, a tendency for some rscFv fragments to accumulate as insoluble proteins in the periplasm, probably due to the aggregation of folding intermediates. Aggregation of folding intermediates competes with correct folding, and is often the limiting factor in the yield of soluble antibody fragment [23].

Even though cytoplasm has a much larger space than periplasm, there was no rscFv band detected from soluble cytoplasmic proteins fraction. This result indicates that the majority of rscFv proteins in the cytoplasm were in the form of inclusion bodies. Cytoplasmic space is kept under reducing conditions by the thioredoxins with the help of thioredoxin reductase and the glutaredoxins with the small molecule glutathione and glutathione reductase. As a result, disulfide bonds cannot occur in this compartment [24]. Theoretically, it could be the cause of inclusion bodies formation within the cytoplasm.

From the analysis conducted, it was shown that solubilized rscFv inclusion bodies has a thicker band compared to those from soluble cytoplasmic and periplasmic proteins fraction, and also from extracellular proteins (Fig. 5A). This result was corresponded with previous analysis that rscFv proteins expression in cytoplasmic space were in the form of inclusion bodies.

Extracellular proteins were analyzed by concentrating cell-free culture medium. Based on experience of Kipriyanov et al. [11], some antibody fragments appear to perturb the integrity of the outer membrane and are released into the medium. Recovery from the medium is generally a result of accumulation of the antibody fragment in periplasm causing the cell outer membrane become leaky, leading to leakage of the antibody fragment into the medium [23].

Western blot analysis was performed using His-Probe rabbit polyclonal IgG antibody. Fig. 5B showed 29.81 kDa bands were detected in total proteins, soluble periplasmic proteins, solubilized inclusion bodies, and extracellular proteins. This result proving that the 29.81 kDa proteins found on SDS-PAGE analysis were rscFv proteins which carry the His-tag sequence.

After all of analyses have been conducted, we reported that the pJ414-rscFv plasmid has been successfully constructed and was confirmed by restriction, PCR and DNA sequencing analyses. The result of SDS-PAGE and Western blot analyses showed that V_L_-linker-V_H_ orientation of anti-EGFRvIII scFv were detected in total proteins, soluble periplasmic proteins, solubilized inclusion bodies, and extracellular proteins with a molecular size of approximately 29.81 kDa. In addition, PelB leader sequence has successfully translocated the rscFv proteins into the periplasmic space of *E. coli* Nico21(DE3). Based on current research and previous research [16], we can conclude that both scFv and rscFv can be expressed in *E. coli* expression system.

Our studies support the feasibility of developing method for producing anti-EGFRvIII scFv with reversed orientation. Experiment of Desplancq et al. (15) demonstrated that scFv in the V_L_-linker-V_H_ format showed greater binding activity than those with the V_H_-linker-V_L_. Therefore, for the next study we have to develop the method for evaluating the binding ability, stability and affinity of both scFv and rscFv recognizing EGFRvIII epitope. ScFv which shows greater binding activity can be further developed as an agent for immunology-based cancer therapy or diagnostic purposed.

## Experimental

### Material and Microorganisms

Expression vector containing pelB signal sequence and his-tag (pJ414-scFv) was obtained from previous research [16]. Cloning vector containing DNA fragment encoding V_L_-linker-V_H_ orientation scFv (pTz-rscFv), *E. coli* TOP10 and Nico21(DE3) were available in our laboratory. The primers used for amplification and analysis were: rscFv-F primer containing *Nco*I site (5’-GGCCATGGCTGATATTGTTATGACCCAAACACCATTGTC-3’), rscFv-R primer containing *Cla*1 site (5’-AGCCATGGATCGATAGAACCACCACCAGACG-ACACT-3’), and T7-promoter primer (5’-TAATACGACTCACTATAGGG-3’). All primers were purchased from Integrated DNA Technologies (IDT, USA).

**Fig. 6:**
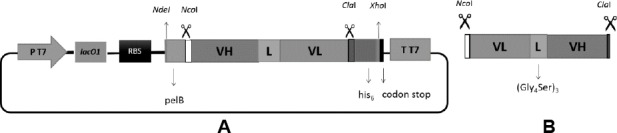
(A) Previous expression vector (pJ414-scFv); (B) rscFv fragment from PCR amplification using pTz-rscFv as template.

### Construction of Plasmid pJ414-rscFv

DNA fragment encoding rscFv proteins was amplified from pTz-rscFv by PCR method using rscFv-F and rscFv-R specific primers. PCR was conducted as follows: initial denaturation at 95°C for 1 m; 25 to 30 cycles of denaturation at 95°C for 1 m, annealing at 62°C for 30 s, and extension at 72°C for 1 m, then final extension at 72°C for 5 m. Purified PCR product and pJ414-scFv expression vector were double digested using *Nco*I and *Cla*I enzymes (3 IU/1 µg product) (Thermo Scientific, USA), then incubated at 37°C for 18 h. Digested products were examined with 1% agarose gel electrophoresis, subsequently isolated from an agarose gel using Gel/PCR DNA Fragments Extraction Kit (Geneaid, Taiwan).

Purified rscFv gene was cloned into expression vector using T4-DNA ligase enzyme (1 IU/50 ng plasmid) (Promega, USA), then transformed into *E. coli* TOP10 using one step transformation and stock solution (TSS) method [25]. Transformants *E. coli* were characterized by colony PCR. Then, the recombinant plasmids from positive colonies were isolated and characterized by restriction and PCR analyses, and subsequently sequenced to ensure no mutation presents within the gene sequence. Confirmed recombinant plasmid was then transformed into *E. coli* Nico21(DE3). Transformants *E. coli* Nico21(DE3) were characterized by colony PCR and total proteins expression. Colony PCR was performed using 2 µL of culture as template and PCR protocol as mentioned above except that 5 minutes of initial denaturation at 95°C was used.

### Growth Condition for rscFv Expression

*E. coli* Nico21(DE3) carrying pJ414-rscFv were grown overnight in Luria Bertani medium with 100 µg/mL ampicillin (LBamp) at 25°C. This culture was diluted 1:50 with LBamp medium, and then grown at 25°C. When cultures reached OD_600_ = 0.8, IPTG was added and growth was continued at 25°C for 16-18 h [26]. Optimization of IPTG concentration was carried out by varying IPTG concentration from 0.1 to 1 mM.

### Isolation of Soluble Periplasmic Proteins

Cells were harvested by centrifugation at 5000 x *g* and 4°C for 15 m. Pelleted cells were resuspended in hypertonic solution of cell lysis buffer (20% sucrose, 30 mM Tris-Cl, and 1 mM EDTA pH 8). After 10 m of incubation on ice with occasional stirring, the cells were centrifuged at 11600 x *g* and 4°C for 20 m. The supernatant was collected and the remaining pellets were resuspended with the same volume of ice-cold 5 mM MgSO_4_ solution, and then incubated on ice for 30 m with gently agitation. The spheroplasts were centrifuged at 11600 x *g* and 4°C for 30 m and the supernatant was collected and combined with the supernatant from hypertonic solution [27].

### Comparison of Total Proteins, Soluble Periplasmic and Cytoplasmic Proteins, Solubilized Inclusion Bodies, and Extracellular Proteins

As much as 100 mL culture was prepared by method previously described, induced by optimized IPTG concentration. Analysis of total proteins was carried out by adding 50 µL of sample buffer and 25 µL of denaturing lysis buffer (8 M urea, 10 mM Tris-Cl, and 100 mM NaH_2_PO_4_) into pellet cells from 1 mL culture, and then boiled for 15 m. The remaining culture was pelleted, and then soluble periplasmic and cytoplasmic proteins were isolated.

Isolation of cytoplasmic proteins was performed by freeze-thaw method. Spheroplasts from periplasmic extraction were resuspended in lysis buffer (50 mM Tris-Cl, 3 mM EDTA, and 1 mM PMSF). Spheroplast suspension was subjected to five freeze-thaw cycles by freezing at −20°C for 15 m and thawing for 15 m in room temperature. After that, thawed spheroplast suspension was centrifuged at 11600 x *g* and 4°C for 20 m, leaving the soluble cytoplasmic extract as the supernatant [28, 29].

After soluble cytoplasmic proteins had been isolated, the remaining pellets were resuspended in denaturing lysis buffer. Suspension was incubated on ice with gently agitation for 1h, and then centrifuged at 11600 x *g* and 4°C for 20 m. This supernatant contained solubilized inclusion bodies.

Cell-free culture supernatant was concentrated to analyze the extracellular proteins. As much as 100 µL of trichloroacetic acid was added into 900 µL of cell-free culture supernatant, and incubated at 8°C for 2 h. It was then centrifuged at 11600 x *g* and 4°C for 20 m, and the pellets were washed with 200 µL of acetone [30]. This washing step was repeated two times. Pelleted proteins were resuspended in phosphate buffered saline (300 mM NaCl and 50 mM NaH_2_PO_4_) containing 1% of Triton X-100. Total proteins, soluble cytoplasmic and periplasmic proteins, solubilized inclusion bodies, and also extracellular proteins were then analyzed by 13% SDS-PAGE and Western blot analyses.

### Western Blot Analysis

Briefly, after the proteins had been separated by 13% SDS-PAGE, the gel was immersed in the transfer buffer (25 mM Tris base, 192 mM Glycine, and 20% methanol). The proteins were transferred to a nitrocellulose membrane equilibrated in transfer buffer using Mini Trans-Blot® Cell (Bio-Rad) at 90 V for 2 h. The membrane was incubated in a blocking buffer [5% skim milk in Tris-buffered saline pH 7.6 (50 mM Tris-Cl and 150 mM NaCl)] for 1 h at room temperature and then washed 3 times (10 m each) with TBS-T (0.1% Tween-20 in TBS). The membrane was incubated with His-Probe rabbit polyclonal IgG antibody (1:2000 dilution, Santa Cruz Biotechnology, Inc., USA) overnight at 16°C. After 3 times washes (10 min each) with TBS-T, the membrane was incubated with AP-conjugated goat anti-rabbit IgG antibody (1:3500 dilution, Santa Cruz Biotechnology, Inc., USA) at room temperature for 1 h. The membrane was then washed 3 times with TBS-T. The signals were then visualized with NBT-BCIP (Thermo Scientific, USA).
